# Link prediction based on non-negative matrix factorization

**DOI:** 10.1371/journal.pone.0182968

**Published:** 2017-08-30

**Authors:** Bolun Chen, Fenfen Li, Senbo Chen, Ronglin Hu, Ling Chen

**Affiliations:** 1 College of Computer Engineering, Huaiyin Institute of Technology, Huaian, China; 2 School of Computer Science and Technology, Nanjing University of Aeronautics and Astronautics, Nanjing, China; 3 Department of Computer Science, Yangzhou University, Yangzhou, China; 4 State Key Lab of Novel Software Tech, Nanjing University, Nanjing, China; Hangzhou Normal University, CHINA

## Abstract

With the rapid expansion of internet, the complex networks has become high-dimensional, sparse and redundant. Besides, the problem of link prediction in such networks has also obatined increasingly attention from different types of domains like information science, anthropology, sociology and computer sciences. It makes requirements for effective link prediction techniques to extract the most essential and relevant information for online users in internet. Therefore, this paper attempts to put forward a link prediction algorithm based on non-negative matrix factorization. In the algorithm, we reconstruct the correlation between different types of matrix through the projection of high-dimensional vector space to a low-dimensional one, and then use the similarity between the column vectors of the weight matrix as the scoring matrix. The experiment results demonstrate that the algorithm not only reduces data storage space but also effectively makes the improvements of the prediction performance during the process of sustaining a low time complexity.

## Introduction

With the unprecedentedly rapid development of technology, the world has become increasingly complicated with frequent networking. In the real world, a number of information, biological, and social systems, ranging from interpersonal relationships to the colony structure, from transportation to the online world, from the ecosystem to the nervous system, can be considered as a network, in which vertices stand for the interactions between vertices or links and entities denote relations. Undoubtedly, there will be some potential links which are undetected and meanwhile there will be redundant links or some errors during the process of complicated network due to the limitations of space or time as well as the experimental conditions. Besides, based on the known network information, we are required to forecast the potential and missing links. This is the objectivity of the forecast challenge linked with the network, due to the dynamic development of the links of complicated network [1, 2].

In fact, the link prediction method can be utilized as an auxiliary means to investigate the structure of social network. Actually, it is essential to forecast the potential link of people or the future. There is an extensive scope of practical application values in varieties of areas in the link prediction problem. For example, users’ potential friends can be shown by the prediction of link, and even introduced to others in networks online [3]. Besides, the potential links between people can be found based on the analysis on the social relations [4–6]. Furthermore, the prediction of link could be utilized in the academic network to forecast the cooperators as well as the kind of an article [7]. The link between the nodes shows an interactive relation in the biological networks, such as disease-gene and metabolic networks as well as protein-protein interaction networks [8]. In addition, the research on link prediction has significant theoretical importance and an extensive scope of practical value [9–12]. For instance, it can offer a unified and convenient platform which can compare the mechanisms of network development more fairly and help comprehend the mechanism of the development of complicated networking theoretically, in order to develop the theoretical study on the model of complicated network evolution.

In recent decades, one of the link prediction problems of increasing interest revolves around the expansion of network size, the scoring matrix sparsely and the noise of the data. Due to high-dimensionality of user-rating matrix, the time and space complexity of personalized link prediction are increasing, which will cause a great impact on the performance of the prediction system. To deal with this problem, numerous new methods have been reported to improve the efficiency of link prediction. For example, Liu and Lv [13]used the local random walk instead of the global random walk to solve the link prediction problem, which can achieve good result. In [14], G.Rossetti et al defined the concept the multidimensional link prediction problem and proposed the several predictors based on node correlation. H. H. Song et al. [15] considered that the new edges would be linked in the near future and proposed the incremental update algorithm to deal with the large-scale networks.

In addition, with the consideration of the importance of network organization principle, Pan [16] proposed a predefined structural Hamiltonian algorithm which can be used to calculate the probability of non-observed links. Liu et al [17] proposed a local naive Bayes model based on the fact that various neighbors might have various functions, thus resulting in various outcomes. Hu [18] put forward a new model that examines the performances of the algorithms of state-of-the-art recommendation in the constantly developing network. Besides, it was also found that with the passage of time, the accuracy of recommendation gradually reduces if the online network evolution completely depends on the recommendation. In order to optimize the weights applied in a linear combination of node similarity indices and sixteen neighborhood, a method is provided by Bliss to predict the links in the future with the application of Covariance Matrix Adaptation Evolution Strategy (CMA-ES) [19]. Barzel B [20] used global silencing of indirect correlations to make the prediction of missing links. A global algorithm of optimization is presented by Zhu which can infer the potential space effectively. Two alternative algorithms of optimization with incremental and local updates are also put forward to make the model scale to the bigger network without compromising the accuracy of forecast [2]. A nonparametric method has been proposed to link prediction in large-scale dynamic networks by Sarkar [21]. They considered that the characteristics of pairs of nodes based on graph and those of the local neighborhoods are utilized through using the model to forecast whether the nodes can be linked at every step of time. Richard [22] proposed a vector autoregressive (VAR) model based on the node degree to enhance the forecast accuracy. Zhang et al [23]proposed a link prediction algorithm based on an integrated diffusion on user-item-tag tripartite graphs and found that the usage of tag information can significantly improve novelty of link prediction. Recently, some swarm intelligence methods are also involved in link prediction. For example, Sherkat [24] proposed a new unsupervised structural ant colony approach algorithm which has the best outcome in some networks. Despite that those methods are designed especially for the large scale networks, the accuracy of the results cannot be guaranteed due to the limit of computation time [25].

Nowadays, matrix decomposition technology has been widely applied, because it is relatively simple and able to obtain high prediction accuracy [26]. There are numerous well-known matrix decomposition methods, such as: singular value decomposition (SVD) [27], principal component analysis (PCA) [28, 29], independent component analysis (ICA) [30–32]. In SVD, it requires complementing scoring matrix in order to solve the sparsity of the network, but the operation would seriously increase the data storage space, therefore and it is not practical in the link prediction systems. Furthermore, due to the high time complexity of SVD, it is not applicable for large-scale network. With the aim to improve the performance of the SVD based method, SimonFunk [33] proposed LFM models. However, in the actual scoring system, the user ratings for goods do not have a uniform standard, which are of large arbitrariness due to the personalized habits on selecting goods. LFM model does not take the impact of the users’ history buying into consideration. To overcome such shortcomings of LFM model, Koren [34] proposed a SVD++ model based on the LFM joined with the user’s history scoring.

However, the existing matrix factorization models do not consider the situation that negative elements exist in the matrix. In real world applications, the a negative score by a user for an object does not necessarily mean that this object will never be selected by the user As time goes on, maybe this user will become interested in this object. Therefore, Lee and Seung [35, 36] proposed a method based on non-negative matrix factorization (NMF). It is a collection of algorithms which depends on linear algebra and multivariate analysis where two matrices without any negative elements consist in the original matrix. It is because of this non-negativity that makes it easier to analysis the final matrices. Therefore, NMF technology has been widely used in data mining [37, 38], image analysis [39–41], medical imaging [42] and voice processing [43], etc. However, in the link prediction, it has not attracted broad attentions. In this paper, we will apply NMF technology in link prediction and propose the NMF-LP model.

## Materials and methods

### Problem formulation and evaluation methods

Using an undirected and simple network which has node attitudes *G*(*U*, *V*, *E*), in which *E* means the series of links, *U* refers to the series of nodes, and *V* is the series of node attitudes can represent a network. *G* does not allow various self-connections and links. Let *M* = |*V*| be the amount of node attitudes and *N* = |*U*| be the amount of nodes in *G*. *U* is considered to represent the universal set which all the possible links of *N*(*N* − 1)/2 are consisted in. Link prediction aims to identify the links which will occur in the future or the missing links in the series of nonexistent links *U* − *E*.

Assigning *S*(*x*, *y*) as a score which stands for the similarities between the two nodes to every pair of the nodes (*x*, *y*) ∈ *U* is our method. In a pair of nodes, (*x*, *y*) in *U*\*E*, larger *S*(*x*, *y*) tends to lead to higher possibility in a link between the nodes.

To test the accuracy of the results by our algorithm, the observed links in *E* are randomly divided into two parts: the training set, *E*^*T*^, which is treated as known information, while the probe set (i.e., validation subset), *E*^*P*^, which is used for testing and no information in this set is used for prediction. *ET* ∪ *EP* = *E* and *ET* ∩ *EP* = ∅. In principle, a link prediction algorithm provides an ordered list of all non-observed links (i.e., *U* − *E*^*T*^) or equivalently gives each non-observed link, say (*x*, *y*) ∈ *U* − *E*^*T*^, a score *S*_*xy*_ to quantify its existence likelihood. To evaluate the accuracy of prediction algorithms, there are two standard metrics: *AUC* and *Precision*.

(1) *AUC*

The value of AUC is the area under the curve of ROC(receiver operating characteristic). we randomly choose a missing link and nonexistent link. After doing *n* times’ independent evaluations, there should have *n*′′ times’ missing link which shows a similar score and a higher score. Under this situation, the value of *AUC* is as below:

$$\begin{array}{c} AUC=\frac{n\prime +0.5n\prime \prime }{n} \end{array}$$ (1)

Generally, a bigger value of AUC shows better performance; thus, the AUC result through a randomly selected predictor is 0.5 while the AUC value of the perfect result is 1.0.

(2) *Precision*

Given the ranking of the non-observed links, the *Precision* is defined as the ratio of relevant objects selected to the total number of objects selected. That is to say, if we take the Top-*L* links as the predicted ones, among which *m* links are right, then the *Precision* can be expressed as:

$$\begin{array}{c} Precision=\frac{m}{L} \end{array}$$ (2)

Clearly, higher precision means higher prediction accuracy.

### Non-negative matrix factorization and link prediction

For the reader’s reference, Table 1 summarizes frequently used notations.

**Table 1:** Notations and their meanings.

Notation	Meaning
*A*	adjacent matrix of the network
*B*	attribute similarity matrix of the network
*U*	*U* consists of the bases of the latent space, and is called the base matrix of *A*
*V*	*V* represents the combination coefficients of the bases for reconstructing the matrix *A*, and is called the coefficient matrix.
*U*^(*B*)^	*U*^(*B*)^ consists of the bases of the latent space, and is called the base matrix of *B*
*V*^(*B*)^	*V*^(*B*)^ represents the combination coefficients of the bases for reconstructing the matrix *B*, and is called the coefficient matrix.
*Q*, *Q*^(*B*)^	*Q*, *Q*^(*B*)^ are auxiliary diagonal matrixes
*F*, *G*	*Q*, $F\in R_{+}^{n*m}$ and $G\in R_{+}^{k*k}$ are auxiliary diagonal matrixes
*ε*	error between the original matrix and the product of the base matrix and weight matrix

Here $A\in R_{+}^{n*n}$ and $B\in R_{+}^{n*m}$, where *m* is the number of attributes. The values of *B* can be defined within the range [0,1] by normalizing its row vectors. We assume the adjacency matrix *A* as a non-negative characteristic matrix where each column represents the characteristic vector of a vertex and the goal of NMF is to obtain two non-negative matrices *U* ∈ *P*_n*k_ and *V* ∈ *P*_k*n_ so that their product is very close to matrix *A*:

$$\begin{array}{c} A=UV \end{array}$$ (3)

Here, *k* is the dimension of the latent space (*k* < *n*). *U* consists of the bases of the latent space, and is called the base matrix. *V* represents the combination coefficients of the bases for reconstructing the matrix *A*, and is called the coefficient matrix. Generally, this decomposition problem can be modeled as the following Frobenius norm optimization problem:

$$\begin{array}{c} \min_{u,v}{∥A-UV∥}_{F}^{2}\;s.t.U\geq 0,V\geq 0 \end{array}$$ (4)

Here, ∥⋅∥_*F*_ is the Frobenius norm, constrain *U* ≥ 0 and *V* ≥ 0 requires that all the elements in matrixes *U* and *V* are nonnegative. The similarity between nodes *i* and *j* in the latent space can be represented by the similarity between the *i*^*th*^ and *j*^*th*^ row vectors in matrix *V*. Similarly, NMF form of the attribute matrix *B* is as follows:

$$\begin{array}{c} B=U^{\left(B\right)}V^{\left(B\right)} \end{array}$$ (5)

Therefore, we need to solve the optimization problem as follows:

$$\begin{array}{c} \min_{u^{\left(B\right)},v^{\left(B\right)}}{∥B-U^{\left(B\right)}V^{\left(B\right)}∥}_{F}^{2}\;s.t.U^{\left(B\right)}\geq 0,V^{\left(B\right)}\geq 0 \end{array}$$ (6)

Here, *U*^(*B*)^ ≥ 0 and *V*^(*B*)^ ≥ 0 are respectively *n* * *k* and *k* * *m* non-negative matrixes.

In order to integrate the topology matrix *A* and the attribute matrix *B*, we map them into a latent space by a same projection. Such mapping can be implemented by non-negative matrix factorizings on *A* and *B*. However, when we factorize *A* and *B* at the same time, we cannot ensure that the projection matrixes *V* and *V*^(*B*)^ are identical. Therefore, we use matrix V* to make *V* and *V*^(*B*)^ has the minimal distance between *V* and *V*^(*B*)^, namely, we need to minimize the formula as follows:

$$\begin{array}{c} {\lambda ∥V-V^{*}∥}_{F}^{2}+{\mu ∥V^{\left(B\right)}-V^{*}∥}_{F}^{2}\;s.t.U^{\left(B\right)}\geq 0,V^{B}\geq 0. \end{array}$$ (7)

Therefore, our goal is to solve the following the optimization problem:

$$\begin{array}{c} \begin{array}{c} \min_{u,u^{\left(B\right)},v,v^{\left(B\right)}}{∥A-UV∥}_{F}^{2}+{∥B-U^{\left(B\right)}V^{\left(B\right)}∥}_{F}^{2}+{\lambda ∥V-V^{*}∥}_{F}^{2}+{\mu ∥V^{\left(B\right)}-V^{*}∥}_{F}^{2}\\ s.t.{∥U∥}_{1}=1,{∥U^{B}∥}_{1}=1,U\geq 0,V\geq 0,U^{\left(B\right)}\geq 0,V^{\left(B\right)}\geq 0. \end{array} \end{array}$$ (8)

In order to remove the constrains ∥*U*∥_1_ = 1 and ∥*U*^(*B*)^∥_1_ = 1 in Eq (8), we define auxiliary diagonal matrixes *Q* and *Q*^(*B*)^ as follows:

$$\begin{array}{c} Q=diag(\Sigma_{i}u_{i1},\Sigma_{i}u_{i2},\ldots ,\Sigma_{i}u_{ik}) \end{array}$$ (9)

$$\begin{array}{c} Q^{\left(B\right)}=diag\left(\Sigma_{i}u_{i1}^{\left(B\right)},\Sigma_{i}u_{i2}^{\left(B\right)},\ldots ,\Sigma_{i}u_{ik}^{\left(B\right)}\right) \end{array}$$ (10)

The matrixes *U* and *U*^(*B*)^ can be normalized into *UQ*^−1^ and $U^{\left(B\right)}Q^{\left(B\right)}{}^{{}^{-1}}$. Since *UV* = (*UQ*^−1^)(*QV*) and $U^{\left(B\right)}V^{\left(B\right)}=(U^{\left(B\right)}Q^{\left(B\right)}{}^{{}^{-1}})(Q^{\left(B\right)}V^{\left(B\right)})$, the matrixes *V* and *V*^(*B*)^ can be normalized into *VQ* and *V*^(*B*)^*Q*^(*B*)^ respectively. Therefore the optimal problem (8) is equivalent to the one as follows:

$$\begin{array}{c} \min_{u,u^{B},v,v^{B}}J(u,u^{\left(B\right)},v,v^{\left(B\right)},v^{*})\;s.t.U\geq 0,V\geq 0,U^{B}\geq 0,V^{B}\geq 0. \end{array}$$ (11)

Here,

$$\begin{array}{c} J\left(u,u^{\left(B\right)},v,v^{\left(B\right)},v^{*}\right)=∥A-UV{∥}_{F}^{2}+∥B-U^{\left(B\right)}V^{\left(B\right)}{∥}_{F}^{2}\\ \begin{array}{c} +\lambda ∥QV-V^{*}{∥}_{F}^{2}+\mu ∥Q^{\left(B\right)}V^{\left(B\right)}-V^{*}{∥}_{F}^{2} \end{array} \end{array}$$

Therefore, our goal is to find the optimal resolution of the formula (11).

### Iterative update rule of non-negative matrix

To solve the optimization problem in Eq (11), we present an iterative method. Since *U*, *V*, *U*^(*B*)^, *V*^(*B*)^ and V* are variables in Eq (11), we fix four of the variables at each time in the iteration, and obtain the best value the fifth variable to minimize the objective function *J*. After setting proper initial values for matrices *U*, *V*, *U*^(*B*)^, *V*^(*B*)^ and V*, each iteration consists of five steps: a. Fix the matrices *V*, *U*^(*B*)^, *V*^(*B*)^ and V*, update *U* to minimize *J*; b. Fix the matrices *U*, *U*^(*B*)^, *V*^(*B*)^ and V*, update *V* to minimize *J*; c. Fix the matrices *U*, *V*, *V*^(*B*)^ and V*, update *U*^(*B*)^ to minimize *J*; d. Fix the matrices *U*, *V*, *U*^(*B*)^ and V*, update *V*^(*B*)^ to minimize *J*; e. Fix the matrices *U*, *V*, *U*^(*B*)^ and *V*^(*B*)^, update V* to minimize *J*.

The updating steps above are repeated until convergence.

#### Updating *U*

Fixing the matrices *V*, *U*^(*B*)^, *V*^(*B*)^ and V*, minimizing *J* by updating *U* is equivalent to minimize the objective function:

$$\begin{array}{c} F\left(U\right)={∥A-UV∥}_{F}^{2}+\lambda {∥QV-V^{*}∥}_{F}^{2} \end{array}$$ (12)

**Derivation 1** To optimize the objective function (12), the following updating rule can be used:

$$U_{lm}=U_{lm}\frac{\sum_{j}A_{ij}V_{mj}+\lambda \sum_{j}V_{mj}^{*}V_{mj}}{\lambda \sum_{j}V_{mj}^{2}\sum_{h}V_{hm}+\sum_{j}V_{mj}\sum_{k}U_{lk}V_{kj}}$$ (13)

**Proof**:

According to the definition of Frobenius norm definition, we can get:

$$\begin{array}{ll} F\left(U\right) & =\sum_{i}\sum_{j}A_{ij}-\sum_{k}U_{ik}V_{kj}{}^{2}+\lambda \sum_{i}\sum_{j}\sum_{k}Q_{ik}V_{kj}-V_{ij}^{*}{}^{2}\\ & \begin{array}{c} =\sum_{i}\sum_{j}\left[A_{ij}^{2}-2A_{ij}^{2}\sum_{k}U_{ik}V_{kj}+{\left(\sum_{k}U_{ik}V_{kj}\right)}^{2}\right] \end{array}\\ & \begin{array}{c} +\lambda \sum_{i}\sum_{j}\left[{\left(\sum_{k}Q_{ik}V_{kj}\right)}^{2}-2V_{ij}^{*}\sum_{k}Q_{ik}V_{kj}+{\left(V_{ij}^{*}\right)}^{2}\right] \end{array} \end{array}$$

We take the derivative of *F*(*U*) on *U*_*lm*_:

$$\begin{array}{c} \frac{\partial F(U)}{\partial U_{lm}}=-2\sum \left(A_{ij}V_{mj}\right)+2\sum jV_{mj}\sum_{k}U_{lk}V_{kj}+2\lambda \sum jV_{mj}^{2}\sum_{h}U_{hm}-2\lambda \sum_{j}V_{mj}^{*}V_{mj} \end{array}$$

By Karush-Kuhn-Tucker (KKT) condition we know that $\frac{\partial F(U)}{\partial U_{lm}}=0$, and can get:

$$\begin{array}{c} -2\sum_{j}\left(A_{ij}V_{mj}\right)+2\sum_{j}V_{mj}\sum_{k}U_{lk}V_{kj}+2\lambda \sum_{j}V_{mj}^{2}\sum_{h}U_{hm}-2\lambda \sum_{j}V_{mj}^{*}V_{mj}=0 \end{array}$$

Namely:

$$\begin{array}{c} \sum_{j}\left(A_{ij}V_{mj}\right)+\lambda \sum_{j}V_{mj}^{*}V_{mj}=\sum_{j}V_{mj}\sum_{k}U_{lk}V_{kj}+\lambda \sum_{j}V_{mj}^{2}\sum_{h}U_{hm} \end{array}$$

So, the update rule of *U* is as follows:

$$U_{lm}=U_{lm}\frac{\sum_{j}A_{ij}V_{mj}+\lambda \sum_{j}V_{mj}^{*}V_{mj}}{\lambda \sum_{j}V_{mj}^{2}\sum_{h}V_{hm}+\sum_{j}V_{mj}\sum_{k}U_{lk}V_{kj}}$$

#### Updating *U*^(*B*)^

**Derivation 2** we construct the function as follows:

$$\begin{array}{c} F\left(U^{\left(B\right)}\right)={∥A-U^{\left(B\right)}V^{\left(B\right)}∥}_{F}^{2}+\mu {∥QV^{\left(B\right)}-V^{*}∥}_{F}^{2} \end{array}$$ (14)

the update rules for *F*(*U*^(*B*)^) can be written as:

$$U_{lm}^{\left(B\right)}=U_{lm}^{\left(B\right)}\frac{\sum_{j}B_{ij}V_{mj}^{\left(B\right)}+\mu \sum_{j}V_{mj}^{*}V_{mj}^{\left(B\right)}}{\mu \sum_{j}{\left(V_{mj}^{\left(B\right)}\right)}^{2}\sum_{h}U_{hm}^{\left(B\right)}+\sum_{j}U_{mj}^{\left(B\right)}\sum_{k}U_{lk}V_{kj}}$$ (15)

Derivation 2 can be proved in a similar way as Derivation 1.

#### Updating *V*

**Derivation 3** we construct the function as follows:

$$\begin{array}{c} G\left(V\right)={∥A-UV∥}_{F}^{2}+\lambda {∥V-V^{*}∥}_{F}^{2} \end{array}$$ (16)

the update rules for *G*(*V*) can be written as:

$$V_{lm}=V_{lm}\frac{\sum_{i}A_{im}U_{il}+\lambda \sum_{j}V_{lm}^{*}}{\lambda \sum_{i}U_{il}\sum_{k}U_{ik}V_{km}+\lambda V_{lm}}$$ (17)

**Proof**:

According to the definition of Frobenius norm definition, we can get:

$$\begin{array}{ll} G\left(V\right) & =\sum_{i}\sum_{j}A_{ij}-\sum_{k}U_{ik}V_{kj}{}^{2}+\lambda \sum_{i}\sum_{j}V_{ij}-V_{ij}^{*}{}^{2}\\ & \begin{array}{c} =\sum_{i}\sum_{j}\left[A_{ij}^{2}-2A_{ij}^{2}\sum_{k}U_{ik}V_{kj}+{\left(\sum_{k}U_{ik}V_{kj}\right)}^{2}\right] \end{array}\\ & \begin{array}{c} +\lambda \sum_{i}\sum_{j}V_{ij}-V_{ij}^{*}{)}^{2} \end{array} \end{array}$$

We take the derivative of *G*(*V*) on *V*_*lm*_:

$$\begin{array}{c} \frac{\partial G(V)}{\partial V_{lm}}=-2\sum_{i}\left(A_{im}V_{il}\right)+2\sum iU_{il}\sum_{k}U_{ik}V_{km}+2\lambda \left(V_{lm}-V_{lm}^{*}\right) \end{array}$$

By Karush-Kuhn-Tucker (KKT) condition we know that $\frac{\partial G(V)}{\partial V_{lm}}=0$, and can get:

$$\begin{array}{c} -2\sum_{i}\left(A_{im}V_{il}\right)+2\sum iU_{il}\sum_{k}U_{ik}V_{km}+2\lambda \left(V_{lm}-V_{lm}^{*}\right)=0 \end{array}$$ (18)

Namely:

$$\begin{array}{c} \sum_{i}\left(A_{im}U_{il}\right)+\lambda V_{lm}^{*}=\sum_{i}U_{il}\sum_{k}U_{ik}V_{km}+\lambda V_{lm} \end{array}$$

So, the update rule of *V*_*lm*_ is as follows:

$$V_{lm}=V_{lm}\frac{\sum_{i}A_{im}U_{il}+\lambda V_{lm}^{*}}{\sum_{i}U_{il}\sum_{k}U_{ik}V_{km}+\lambda V_{lm}}$$

#### Updating *V*^(*B*)^

**Derivation 4** we construct the function as follows:

$$\begin{array}{c} G\left(V^{\left(B\right)}\right)={∥A-U^{\left(B\right)}V^{\left(B\right)}∥}_{F}^{2}+\mu {∥V^{\left(B\right)}-V^{*}∥}_{F}^{2} \end{array}$$ (19)

the update rules for *G*(*V*^(*B*)^) can be written as:

$$V_{lm}^{\left(B\right)}=V_{lm}^{\left(B\right)}\frac{\sum_{i}B_{im}U_{il}^{\left(B\right)}+\mu V_{lm}^{*}}{\sum_{i}U_{il}^{\left(B\right)}\sum_{k}U_{ik}^{\left(B\right)}V_{km}^{\left(B\right)}+\mu V_{lm}^{\left(B\right)}}$$ (20)

Derivation 4 can be proved in a similar way as Derivation 3.

#### Updating V*

**Derivation 5** we construct the function as follows:

$$\begin{array}{c} H\left(V^{*}\right)={∥QV-V^{*}∥}_{F}^{2}+{∥Q^{\left(B\right)}V^{\left(B\right)}-V^{*}∥}_{F}^{2} \end{array}$$ (21)

the update rules for *H*(V*) can be written as:

$$\begin{array}{c} V_{lm}^{*}=\frac{1}{2}\left(Q_{ll}V_{lm}+Q_{ll}^{\left(B\right)}V_{lm}^{\left(B\right)}\right) \end{array}$$ (22)

**Proof**:

According to the definition of Frobenius norm definition, we can get:

$$\begin{array}{c} H\left(V^{*}\right)=\sum_{i}\sum_{j}{\left(\sum_{k}V_{ik}Q_{kj}-V_{lm}^{*}\right)}^{2}-\sum_{i}\sum_{j}{\left(\sum_{k}V_{ik}^{\left(B\right)}Q_{kj}^{\left(B\right)}-V_{ij}^{*}\right)}^{2} \end{array}$$

We take the derivative of *H*(V*) on $V_{lm}^{*}$:

$$\begin{array}{c} \frac{\partial V^{*}}{\partial V_{lm}^{*}}=-2\left(Q_{ll}V_{lm}-V_{lm}^{*}\right)-2\left(Q_{ll}^{\left(B\right)}V_{lm}^{\left(B\right)}-V_{lm}^{*}\right) \end{array}$$

By Karush-Kuhn-Tucker (KKT) condition we know that $\frac{\partial H(V^{*})}{\partial V_{lm}^{*}}=0$, and can get:

$$\begin{array}{c} Q_{ll}V_{lm}+Q_{ll}^{\left(B\right)}V_{lm}^{\left(B\right)}=2V_{lm}^{*} \end{array}$$

So, we can get the update rule of $V_{lm}^{*}$ as follows:

$$\begin{array}{c} V_{lm}^{*}=\frac{1}{2}\left(Q_{ll}V_{lm}+Q_{ll}^{\left(B\right)}V_{lm}^{\left(B\right)}\right) \end{array}$$

### Framework of the NMF-LP algorithm for link prediction

Based on the iterative method for NMF computing, we present an algorithm named NMF-LP for link prediction based on non-negative matrix factorization. The framework of our algorithm NMF-LP is as follows.

**Algorithm:** NMF-LP (Non-negative Matrix Factorization based Link Prediction)

**Input:** *A*: Adjacency matrix of network;

*B*: Attitude matrix of network;

**Output:** *S*: Scoring matrix between the nodes;

**Begin:**

1. Matrix *A*, *B* regularization

2. Parameter initialization:

3. Set the initial values of *U*, *U*^(*B*)^, *V*, *V*^(*B*)^, V*;

4. **Repeat**

5.   Update *U* according to formula (13);

6.   Calculate the the main diagonal elements of *Q* according to *U*;

7.   Update *U*^(*B*)^ according to formula (15);

8.   Update *V* according to formula (17);

9.   Update *V*^(*B*)^ according to formula (20);

10.   Update V* according to formula (22);

11. **Until** ${∥A-UV∥}_{F}^{2}\;\leq \;\varepsilon$

12. Calculate the similarity between the column vectors of V*;

13. Output the Scoring matrix S between the nodes;

**End**

Line 12 of the algorithm calculates the similarity between the column vectors of weight matrix V*, and store the similarities in the the scoring matrix *S*, which is output on line 13 as the final result of link prediction.

### Time complexity analysis

In each iteration, steps 5 to 10 require *O*(*n*^2^*k*) time. Since each row in matrix V* is a *k*-dimensional vector, which takes *O*(*k*) time to compute the similarity between such vectors. Therefore, step 12 requires *O*(*n*^2^*k*) time for compute the similarities for all pairs of the row vectors in V*. Since *k* and *t* can be treated as constants, complexity of the algorithm is *O*(*n*^2^). In the similarity based link prediction methods, *n*^2^ similarity scores between the node pairs must be computed. Therefore, *O*(*n*^2^) is the lower bound the time complexity of the similarity based link prediction methods.

### Convergence analysis of the algorithm NMF-LP

In this section, we will prove the convergence and correctness of *U*, *V*, *U*^(*B*)^, *V*^(*B*)^ and V* in their iterative process. In order to prove convergence of the algorithm, we will make use of an auxiliary function based on the following lemma:

**Lemma 1** Let $F\in R_{+}^{n\times n}$, $G\in R_{+}^{k\times k}$ be two symmetric matrixes, $S\in R_{+}^{n\times k}$ and $S^{{}^{\prime }}\in R_{+}^{n\times k}$ be two *n* × *k* matrixes. Then we can get:

$$\begin{array}{c} \sum_{i=1}^{n}\sum_{j=1}^{k}\frac{{\left(FS^{{}^{\prime }}G\right)}_{ij}S_{ij}^{2}}{S_{ij}^{{}^{\prime }}}\geq Tr\left[SF^{T}SG\right] \end{array}$$ (23)

**Proof**:

We set $S_{ij}=S_{ij}^{{}^{\prime }}p_{ij}$, then the difference value between the two sides of the formula (22) is:

$$\begin{array}{c} \Delta =\sum_{i,x=1}^{n}\sum_{j,y=1}^{k}F_{ix}S_{xy}^{{}^{\prime }}G_{yj}S_{ij}^{{}^{\prime }}\left(p_{ij}^{2}-p_{ij}p_{xy}\right) \end{array}$$

Noticing that *F*, *G* are symmetric matrixes, we get:

$$\begin{array}{l} \Delta =\sum_{i,x=1}^{n}\sum_{j,y=1}^{k}F_{ix}S_{xy}^{\prime }G_{yj}S_{ij}^{\prime }(\frac{p_{ij}^{2}+p_{xy}^{2}}{2}-p_{ij}p_{xy})\\ \;=\frac{1}{2}\sum_{i,x=1}^{n}\sum_{j,y=1}^{k}F_{ix}S_{xy}^{\prime }G_{yj}S_{ij}^{\prime }{\left(p_{ij}+p_{xy}\right)}^{2}\\ \;\geq 0 \end{array}$$

Namely:

$$\begin{array}{c} \sum_{i=1}^{n}\sum_{j=1}^{k}\frac{{\left(FS^{{}^{\prime }}G\right)}_{ij}S_{ij}^{2}}{s_{ij}^{{}^{\prime }}}\geq Tr\left[SF^{T}SG\right] \end{array}$$

**Theorem 1** is an auxiliary function for *L*(*H*) if the conditions

$$\begin{array}{c} Z\left(H,\tilde{H}\right)\geq L\left(H\right),Z\left(H,H\right)=L\left(H\right) \end{array}$$ (24)

are satisfied.

**Lemma 2** If *Z* is an auxiliary function, then *L* is nonincreasing under the update rule:

$$\begin{array}{c} H\left(\tau +1\right)=arg\min_{H}Z(H,H(\tau )) \end{array}$$ (25)

Based on the above thoughts of constructing auxiliary functions, we give the following lemma to prove the convergence of the algorithm NMF-LP.

**Lemma 3** Fixing any four matrices in *U*, *V*, *U*^(*B*)^, *V*^(*B*)^ and V*, and using the updating rules (13), (15), (17), (20) and (22) in each iteration of algorithm NMF-LP the value of objective function J is non-increasing.

**Proof**:

Firstly, we fix the value of *U*, *U*^(*B*)^, *V*^(*B*)^ and V* to update *V*, we can translate formula (12) into the following optimization problem:

$$\begin{array}{c} J\left(V\right)={∥A-UV∥}_{F}^{2}+\lambda {∥V-V^{*}∥}_{F}^{2} \end{array}$$ (26)

We can get the following equation according to the definition of Frobenius norm:

$$\begin{array}{c} \begin{array}{c} J\left(V\right)=tr\left(-2AUV+V^{T}U^{T}UV\right)+tr\left(\lambda VV^{T}-2\lambda V^{*}V\right)\\ =tr\left(V^{T}U^{T}UV+\lambda VV^{T}\right)-tr\left(2AUV+2\lambda V^{*}V\right) \end{array} \end{array}$$ (27)

We construct the following function:

$$\begin{array}{c} F\left(V^{{}^{\prime }},V\right)=\sum_{jk}\frac{{\left(U^{T}UV+\lambda V\right)}_{jk}{\left(V_{jk}^{{}^{\prime }}\right)}^{2}}{V_{jk}}-\sum_{jk}2{\left(AU+\lambda V^{*}\right)}_{jk}V_{jk}\left(1+\log\frac{V_{jk}^{{}^{\prime }}}{V_{jk}}\right) \end{array}$$ (28)

Then we prove *F*(*V*′, *V*) is an auxiliary function of *J*(*V*). First, when *V*′ = *V*, it is obvious *F*(*V*, *V*) = *J*(*V*). If *V*′ ≠ *V*, and $\exists \frac{V_{ik}^{{}^{\prime }}}{V_{ik}}>0$, we get:

$$\begin{array}{c} \sum_{jk}\frac{{\left(U^{T}UV+\lambda V\right)}_{jk}{\left(V_{jk}^{{}^{\prime }}\right)}^{2}}{V_{jk}}>tr\left(V^{T}U^{T}UV+\lambda VV^{T}\right) \end{array}$$

because of $\frac{V_{ik}^{{}^{\prime }}}{V_{ik}}\geq 1+\log\left(\frac{V_{ik}^{{}^{\prime }}}{V_{ik}}\right)$, $\forall \frac{V_{ik}^{{}^{\prime }}}{V_{ik}}>0$, we know:

$$\begin{array}{c} \sum_{jk}2{\left(AU+\lambda V^{*}\right)}_{jk}V_{jk}\left(1+\log\frac{V_{jk}^{{}^{\prime }}}{V_{jk}}\right)<tr\left(2AUV^{T}+2\lambda V^{*}V\right) \end{array}$$

Therefore, we get the following inequality:

$$\begin{array}{c} F\left(V^{{}^{\prime }},V\right)\geq J\left(V^{{}^{\prime }}\right) \end{array}$$

Therefore *F*(*V*′, *V*) is an auxiliary function of *J*(*V*′).

Based on the definition of auxiliary function and Lemma 2, we know that if we find the *V*′ value to reach the local minimum of auxiliary function *F*(*V*′, *V*^|*t*|^), then the value of *J*(*V*) is non-increasing over *t*. Therefore, we find the *V*′ value to minimize *F*(*V*′, *V*^|*t*|^) through fixing *V*^|*t*|^.

In order to obtain the minimum value of function *F*(*V*′, *V*^|*t*|^), according to KKT conditions, we can obatin:

$$\begin{array}{c} \frac{\partial F(V^{{}^{\prime }},V)}{\partial V_{jk}^{{}^{\prime }}}=\left\{\frac{2{\left(U^{T}UV+\lambda V\right)}_{jk}V_{jk}^{{}^{\prime }}}{V_{jk}}-2\left(AU+\lambda V_{jk}^{*}\right)\frac{V_{jk}}{V_{jk}^{{}^{\prime }}}\right\}=0 \end{array}$$ (29)

So, we can get the update following formula:

$$\begin{array}{c} V_{jk}^{{}^{\prime }}=V_{jk}\frac{{\left(AU\right)}_{jk}+\lambda V_{jk}^{*}}{{\left(U^{T}UV\right)}_{jk}+\lambda V_{jk}} \end{array}$$ (30)

Therefore, *J*(*V*^|*t*|^) is non increasing according to update rule (17) and we can get the update formula about *V*_*jk*_ by replacing $V_{jk}^{{}^{\prime }}$ and *V*_*jk*_ by $V_{jk}^{t+1}$ and $V_{jk}^{\left(t\right)}$, respectability. Therefore, using Eq (17) at each iteration of algorithm NMF-LP, the value of objective function *J* is non-increasing.

In a similar way, we can prove that using the rules (13), (15) and (20) at each iteration of algorithm NMF-LP, for updating *U*, *U*^(*B*)^, *V*^(*B*)^ and V*, respectively, the value of objective function *J* is also non-increasing.

Although it is not guaranteed that the process in Algorithm NMF-LP will converge to a global minimum, the end condition of iterations ${∥A-UV∥}_{F}^{2}\;\leq \;\varepsilon$ can ensure that the result matrixes *UV* is an acceptable factorization of *A* and will meet our requirements in solving the problem of link prediction.

Based on Eq (25), we know that the value of *V* by Eq (30) is also non-increasing. Since *V* > 0, it converges. The correctness of the converged solution is assured by the fact that at convergence, from Eq (30), the solution will satisfy

$$\begin{array}{c} -2\sum_{i}\left(A_{im}V_{il}\right)+2\sum iU_{il}\sum_{k}U_{ik}V_{km}+2\lambda \left(V_{lm}-V_{lm}^{*}\right)=0 \end{array}$$

It is the same as the fixed point condition of Eq (18). In a similar way, the convergence and correctness of formula for updating *U*, *U*^(*B*)^, *V*^(*B*)^ and V* can be proved.

## Results

### Test on networks without node attributes

In this section, we testified the reliability of our algorithm NMF-LP on six benchmark data sets which served networks without node attributes: US airport network(USAir), US political blogs(PB) network, coauthor-ships network between scientists(NS), protein-protein interaction network (PPI), electrical resource grid of the western US(Grid) and Internet(INT). For each non-connected network, we figured out the largest connected component. Table1 listed the topological characteristics of these largest ones from these applied networks, where *N*, *M* respectively mean the amount of nodes and links. *NUMC* indicates the number of the components connected within network as well as the size of the largest component. For instance, 1222/2 can be explained as: for this network, there are 2 connected components, while the largest consists of 1222 nodes. In this table, *e* represents the network’s performance, *C* and *r* are clustering and assortative coefficients. *K* represents the degree of average of network.

To estimate the reliability of outputs, a 10-fold cross-validation, which was randomly produced, was applied. For this applied cross-validation, the original nodes were randomly divided into 10 subsets. Among these 10, one subset served as the criterion data for testifying the reliability of our algorithm, while the other 9 subsets served as data for training. The process of cross-validation was then repeated for 10 times. The average of the 10 outcomes obtained from the folds can be taken as a single estimation.

**Fig 1:**
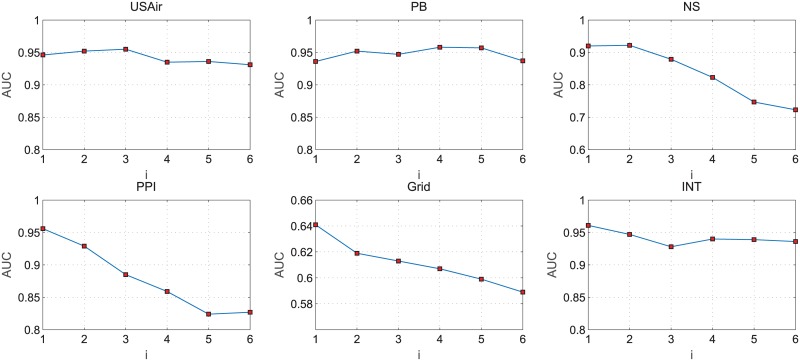
The AUC scores of NMF-LP algorithm with different values of *i*.

In the first step of the non-negative matrix factorization, we need to set the column number of base matrix. We assume that the original adjacency matrix of *N* rows *M* columns, and then the column number (λ) of base vectors are required to satisfy: (*N* + *M*)λ < *NM*. Since *M* = *N* in the network without node attitudes, we can get λ < *N*/2. In our experiments, we set λ = *N*/2^*i*^, where *i* = [1, 2, …, 6]. In the six data sets tested, the changes of the *AUC* scores with different *i* values of NMF-LP algorithm are shown in Fig 1.

From Fig 1, it can be seen that the *AUC* of NMF-LP algorithm shows a decrease trend when the *i* value increases. However it always keeps a high value in six data sets, indicating that NMF-LP algorithm not only can obtain a relatively higher *AUC* value on real networks than other algorithms, but also reduce data storage space.

And we also made comparison between the results produced by NMF-LP and the results of other algorithms such as CN, Jaccard, Sorensen, PA, Salton, HDI, LHN_I and HPI, the criterion applied was *AUC* score. Table 2 listed the averaged *AUC* scores of these tests via NMF-LP and other algorithms. In this table, ‘bold-face’ was applied to mark the highest *AUC* score for each data set through the 9 algorithms.

**Table 2:** The topological features of the giant components of the six networks tested.

Networks	*N*	*M*	*NUMc*	*e*	*C*	*r*	*K*
USAir	332	2126	332/1	0.440	0.749	-0.228	12.807
PB	1224	19090	1222/2	0.397	0.361	-0.079	31.193
NS	1461	2742	379/268	0.016	0.878	0.462	3.754
PPI	2617	11855	2375/92	0.180	0.387	0.454	9.060
Grid	4941	6594	4941/1	0.056	0.107	0.004	2.669
INT	5022	6258	5022/1	0.167	0.033	-0.138	2.492

As can be seen from Table 3, for the 9 algorithms applied in this study, NMF-LP gained the highest *AUC* scores of most of these data sets. Even according to the data set INT, which was considered as the most difficult one, NMF-LP also performed very well, and the score gained was the highest: 0.961. This helped prove the high reliability of the algorithm NMF-LP.

**Table 3:** Comparison of algorithms’ accuracy quantified by AUC.

	USAir	PB	NS	PPI	Grid	INT
CN	0.939	0.926	**0.987**	0.916	0.638	0.650
Salton	0.926	0.878	0.975	0.923	0.612	0.647
Jaccard	0.899	0.865	0.980	0.920	0.622	0.657
Sorensen	0.917	0.885	0.985	0.917	0.633	0.642
HPI	0.840	0.861	0.983	0.910	0.635	0.651
HDI	0.890	0.876	0.980	0.921	0.632	0.652
LHN_I	0.727	0.754	0.972	0.910	0.626	0.650
PA	0.896	0.908	0.671	0.854	0.577	0.959
NMF-LP	**0.955**	**0.958**	0.922	**0.956**	**0.641**	**0.961**

### Test on networks with node attributes

We also testified the reliability of our algorithm NMF-LP on networks with node attributes. We tested 8 sets of data, which can serve as networks. And all of these networks were selected from the website: Digital Bibliography Library Project (DBLP, http://www.informatik.uni-trier.de/ley/db/), a library website for computer science created by University Trier, Germany. It was built in 1980s, and had provided over 2.3 million articles involving computer science. Every significant journal of computer science can be tracked through this website. And also, proceeding papers of important conferences were also recorded by this website. It incorporated lots of collections of publications for CS research. According to our experiment, six datasets were applied, they are: CS Conference (ACM), Complex, Intelligent and Software Intensive Systems (CISIS), International World Wide Web Conferences(WWW), International Conference on Machine Learning (ICML), Applications of Natural Language to Data Bases (NLDB), International Conference on Information and Communication Security (ICICS). We chose some authors from each database. For example, in database ACM, the authors we chose are the people who had been a membership of the ACM conference from 1986 to 1996. We also set a network for every database in which each node was a specific author. And co-authorship between two authors were recorded by the link between the corresponding nodes. And for each author, we could master his overall publications. Key words in the paper titles could represent the attributes of the author. Because networks of some databases were not connected, our tests were only conducted on the largest ones. Table 4 listed the topological characteristics of these largest ones from these applied networks, where *N*, *M* respectively mean the amount of nodes and links. *NUMC* indicates the number of the components connected within network as well as the size of the largest component. And *e* represents the network’s performance, *C* and *r* are clustering and assortative coefficients. *K* represents the degree of average of network.

**Table 4:** The topological features of the giant components of the eight networks tested.

Networks	*N*	*M*	*NUMc*	*e*	*C*	*r*	*K*
ACM	1465	1960	3392/11	0.0014	0.3621	0.5570	3.3010
CISIS	2122	2385	4496/4	0.0078	0.7811	0.1491	7.7766
ICICS	888	1066	1944/3	0.0139	0.7484	0.2726	6.2973
ICML	2640	2213	4843/3	0.0719	0.6470	0.0132	7.2402
NLDB	847	1041	1863/6	0.0072	0.7130	0.2112	5.7190
WWW	5400	3421	1995/897	0.0182	0.7592	0.3724	7.8859

**Fig 2:**
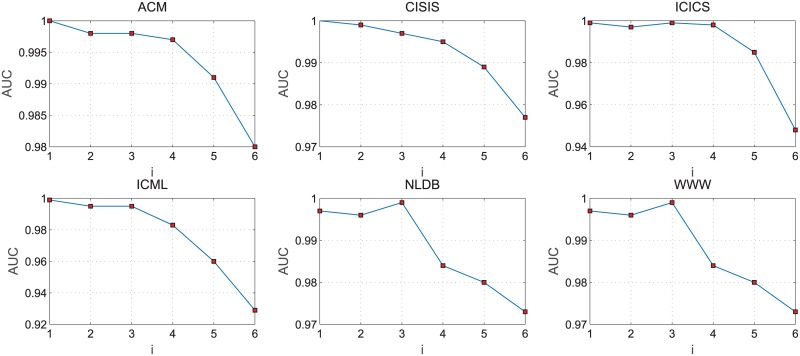
The AUC scores of NMF-LP algorithm with different values of *i*.

In the process of the non-negative matrix factorization, we set λ = (*N* + *M*)/2^*i*+1^, *i* = [1, 2, …, 6], where *N* and *M* are the number of users and attributes. In the eight data sets tested, the changes of the *AUC* scores with different *i* values of NMF-LP algorithm are shown in Fig 2.

Simialr to the test reults on networks without node attitudes, Fig 2 shows that the *AUC* of NMF-LP algorithm presents a decrease trend when the *i* value increases. But it always keeps a high value as i increases in eight data sets. It also indicates that NMF-LP algorithm not only can decompose the scale of the original matrix and reduce dimension of the matrix, but also maintain a relatively higher *AUC* value with reducing the dimension of base vectors.

Apart from that, the AUC scores of the results have also been evaluated and compared by NMF-LP and other algorithms such as Salton, Sorensen, HPI, HDI, LHN_I, PA and CN. 10-fold CV tests which use NMF-LP and other algorithms can test the average *AUC* value, as is shown in Table 4. In the table, what is highlighted in bold-face is the highest *AUC* scores for each data which is set by the 9 algorithms.

In Table 5, we can see that NMF-LP has the highest *AUC* scores on all of the data sets in 9 algorithms. For instance, other algorithms get *AUC* scores less than 0.8635, yet algorithm NMF-LP obtains the highest *AUC* score 0.9980 in dataset ACM, which shows that the algorithm NMF-LP can obtain high quality results that have strong robustness.

**Table 5:** Comparison of algorithms’ accuracy quantified by AUC.

	ACM	CISIS	ICICS	ICML	NLDB	WWW
CN	0.8635	0.9618	0.9598	0.9236	0.9214	0.9505
Salton	0.7932	0.9620	0.9603	0.9238	0.9217	0.9417
Jaccard	0.4223	0.4475	0.3401	0.5223	0.3352	0.4426
Sorensen	0.8552	0.9620	0.9573	0.9236	0.9217	0.9525
HPI	0.8222	0.9621	0.9503	0.9238	0.9216	0.9305
HDI	0.8263	0.9619	0.9602	0.9235	0.9216	0.9505
LHN_I	0.8552	0.9618	0.9342	0.9234	0.9214	0.9504
PA	0.5541	0.5842	0.5335	0.5767	0.5154	0.5846
NMF-LP	**0.9980**	**0.9770**	**0.9850**	**0.9600**	**0.9800**	**0.9600**

### Test on the time requirement of the algorithm

**Fig 3:**
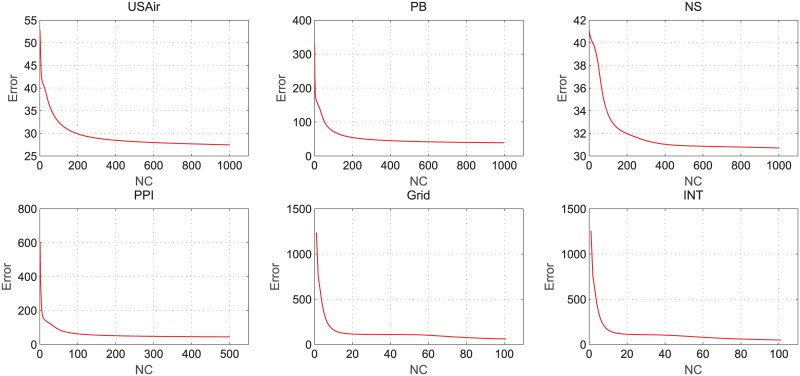
Error changes with the iteration number of NMF on networks without node attitudes.

**Fig 4:**
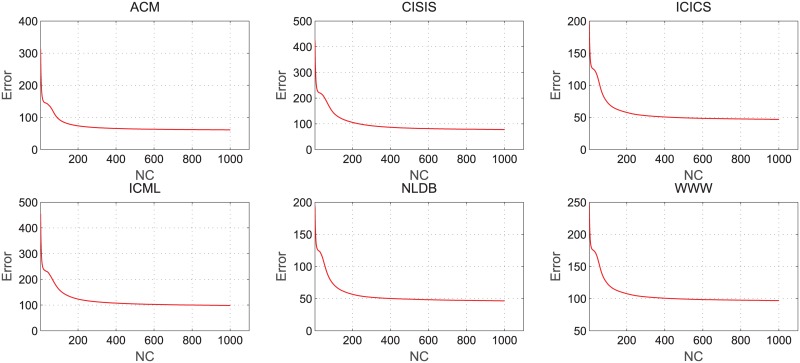
Error changes with the iteration number of NMF on networks with node attitudes.

In the process of the non-negative matrix factorization, we calculate the error between the original matrix *A* and the product of the base matrix *U* and weight matrix *V*. Error formula is $error={∥A-UV|}_{F}^{2}$, we plot the relation between the error and the number of iterations in non-negative matrix factorization on different datasets. The results of tests on networks without node attributes are as shown in Fig 3, and Fig 4 shows the results of tests on networks with node attributes.

Based on Figs 3 and 4, we can see that in the process of non-negative matrix factorization, the tendency of error decreases sharply and it quickly arrives at the condition of convergence. The experiment results demonstrate that the algorithm not only reduces data storage space but also effectively improves the prediction performance while keeping a low time complexity.

## Conclusion

With the expansion of network size, a large amount of redundant information causing by high-dimensionality and sparsely of actual network reduces the performance of the link prediction. In this paper, a new link prediction algorithm was proposed on the basis of non-negative matrix factorization. In the algorithm, the original adjacent and attribute matrixes of the network are effectively decomposed into the base matrix and the weight matrix which are non-negative. Thereafter, we use the similarity of the weight matrix as the scoring matrix between the nodes. The experimental results show that one thing it can gain is higher quality results on real networks comparing with other algorithms, another thing is that it reduces data storage space while maintaining low time complexity.
